# Dietary Phytochemicals, Honey Bee Longevity and Pathogen Tolerance

**DOI:** 10.3390/insects10010014

**Published:** 2019-01-08

**Authors:** Elisa Bernklau, Louis Bjostad, Alison Hogeboom, Ashley Carlisle, Arathi H. S.

**Affiliations:** 1 Bioagricultural Sciences and Pest Management, Colorado State University, Fort Collins, CO 80523, USA; elisa.bernklau@colostate.edu (E.B.); Louis.Bjostad@colostate.edu (L.B.); 2 Department of Soil and Crop Sciences, Colorado State University, Fort Collins, CO 80523, USA; Alison.Hogeboom@colostate.edu; 3 Department of Fisheries, Wildlife and Conservation Biology, Colorado State University, Fort Collins, CO 80523, USA; carlisleashley@ymail.com

**Keywords:** *Apis mellifera*, honey bees, nectar, *Nosema ceranae*, phytochemicals, plant-pollinator interactions

## Abstract

Continued loss of natural habitats with native prairies and wildflower patches is eliminating diverse sources of pollen, nectar and phytochemicals therein for foraging bees. The longstanding plant-pollinator mutualism reiterates the role of phytochemicals in sustaining plant-pollinator relationship and promoting honey bee health. We studied the effects of four phytochemicals—caffeine, gallic acid, kaempferol and *p*-coumaric acid, on survival and pathogen tolerance in the European honey bee, *Apis mellifera* (L.). We recorded longevity of worker bees that were provided *ad libitum* access to sugar solution supplemented with different concentrations of phytochemicals. We artificially infected worker bees with the protozoan parasite, *Nosema ceranae*. Infected bees were provided access to the same concentrations of the phytochemicals in the sugar solution, and their longevity and spore load at mortality were determined. Bees supplemented with dietary phytochemicals survived longer and lower concentrations were generally more beneficial. Dietary phytochemicals enabled bees to combat infection as seen by reduced spore-load at mortality. Many of the phytochemicals are plant defense compounds that pollinators have evolved to tolerate and derive benefits from. Our findings support the chemical bases of co-evolutionary interactions and reiterate the importance of diversity in floral nutrition sources to sustain healthy honey bee populations by strengthening the natural mutualistic relationships.

## 1. Introduction

Recent fluctuations seen in honey bee populations have been attributed to habitat degradation, chemical intensive agriculture, pests and pathogens [1]. Even as the demand for pollination services continues to grow, the ecological impacts of modern agriculture, including the elimination of remnants of native prairies and wildflower patches, are leading to a distinct drop in the diversity of the pollen/nectar diet of bees, compromising their health and physiological abilities [2,3]. Adult bees need continuous access to carbohydrate, protein, lipids and other floral compounds to meet their energy needs and the nutrients in pollen and nectar play a vital role in meeting these needs and improving the ability of bees to cope with stress [4,5,6,7,8].

The relationships between flowering plants and their pollinators have an extensive co-evolutionary history. Embedded within this long-standing plant-pollinator mutualism, is the nutritional benefit for pollinators in return for the pollination services [9]. Honey bees are among the most efficient pollinators, and the floral nectar and pollen they collect offer an assortment of proteins, lipids, carbohydrates, as well as micronutrients such as vitamins and phytochemicals. These primary constituents of nectar and pollen are essential for larval, and adult nutrition [4]. While macronutrients derived from floral rewards play an integral role in the life history stages of honey bees, the role of phytochemicals is only now being explored [10]. Understanding the impact of dietary phytochemicals is necessary to enhance reproduction, development, and foraging activities in honey bees which will pave the way towards maintaining healthy colonies and sustainable populations. Adequate nutrition is essential for normal honey bee colony growth and productivity. Individual bees with adequate and balanced nutrition are better equipped to resist biotic and abiotic stressors and contribute effectively to improving colony food storage and performance [4,5].

Plant-pollinator relationships through the lens of chemical ecology may hold answers to establishing healthy bee populations. Availability and diversity of floral resources strongly influence their nutritional value [11], specifically the balance of carbohydrates, lipids and proteins, and the availability of plant secondary chemicals (phytochemicals) present in nectar and pollen [10,12]. A suitable diet not only satisfies the metabolic needs of individuals, but boosts immunocompetence, and resiliency to pathogens and pesticides [13]. While carbohydrates meet the high metabolic demands of adult bees, lipids serve a multitude of functions including physiological dimorphisms between worker task groups in adult honeybees, and proteins drive the development and growth of larvae and young workers and enhance longevity [14,15,16,17]. Current research suggests that phytochemicals confer benefits against infection [18,19] while improving immune competence and stress tolerance in bees [8,20].

Although the evolution of plant secondary metabolites is rooted in defense from herbivory, pollinators experience both detrimental and beneficial effects from these compounds. Coumarins, flavonols, and alkaloids that are generally toxic, can counteract pathogen infections and enhance physiological function [21,22,23]. While the effects of phytochemicals such as thymol and eugenol are beneficial [24,25,26], the effects of certain phenolic acids, flavonols, flavanones and flavones [13,27,28] on bee health are not fully understood. Phytochemicals, such as thymol, may also result in adverse reactions including loss of phototaxis (adult bees) and vitellogenin expression (larvae) [29]. The persistence of plant-pollinator mutualisms not only reiterates the role of plant chemicals in sustaining this relationship [30,31] but also implies that phytochemicals are likely to be important. Understanding this chemical relationship between bees and plants could be a critical step towards discerning the intricacies of bee health as influenced by the plants. 

Pollen and nectar in flowers offer a diverse array of chemicals [12,22,28,32,33,34,35]. Some of these, including phenolic acids, flavonols [8] and alkaloids [36,37], have been shown to be beneficial to pollinators. In a preliminary experiment, we tested a variety of phytochemicals for their effect on forager longevity. Two of the compounds we tested (phenolic acids including caffeic acid and ferulic acid) had no significant effects, while the other compounds including caffeine (alkaloid), gallic acid and *p*-coumaric acid (phenolic acids) as well as kaempferol (flavonol), significantly increased worker bee longevity. These chemicals (including *p-*coumaric acid where an increase in worker bee longevity has been previously demonstrated [8]) were therefore chosen for further experiments in the current study. Our goal in the current study is to reveal different classes of phytochemicals that are beneficial to honey bees. The responses we measured include worker bee survival and tolerance to *Nosema ceranae* infection when bees received dietary supplementation with the four different phytochemicals—caffeine, gallic acid, kaempferol and *p*-coumaric acid.

## 2. Materials and Methods

Experiments were performed in Fort Collins (CO, USA) with colonies of *Apis mellifera*. For dietary supplementation with phytochemicals, single age-cohort bees were obtained using the standard queen restraining procedure. Queens from experimental colonies were provided with empty, uniquely marked frames to lay their eggs. The queen was caged in an empty frame for 24 h to ensure that all eggs on the frame were being laid during that duration. The date when the queen was caged was marked on the frame. Such marked frames containing late-stage pupae were removed from source colonies after 21 days and placed in an incubator at 32 °C and 50% humidity until the day of emergence [38,39,40]. Adult individuals were color marked on the day of emergence using different colors for different days to indicate unique age cohorts. Marked bees were returned to source colonies, collected after eight days for our assays. We chose the eight-day-old bees as they were middle-aged and had a higher likelihood of being in-hive in comparison to the older bees that were out foraging. This age is also suited for artificial infection with *N. ceranae* spores as compared to the newly emerged bees [41]. The studies described below were conducted during the summer season (May through August) on three colonies each over a two-year period.

### 2.1. Survival Assay

Eight-day-old bees were assigned to single-use cup cages such that there were ten bees in each cage [42]. Bees had *ad libitum* access to feed on the appropriate phytochemical provided as treatments in each cup cage. Treatments consisted of a control (20% sugar solution) and four phytochemicals—caffeine, gallic acid, kaempferol and *p*-coumaric acid, at three concentrations delivered in feeding syringes (Table 1). We aimed to test a wide array of concentrations from low nectar-relevant concentrations to very high, potentially toxic concentrations to determine responses spanning these concentrations. While reports on the nectar concentrations of gallic acid, kaempferol and *p-*coumaric acid are lacking, studies on caffeine demonstrate that 25 ppm is within the natural range [22,36,43]) and 2500 ppm is higher than the reported toxic concentration for caffeine [33]). We chose to test three concentrations—25 ppm, 250 ppm and 2500 ppm for all the four phytochemicals. Test compounds were obtained from Sigma-Aldrich (St. Paul, MN, USA) and were all at least 97.5% purity (kaempferol Cat. No. K0133, *p*-coumaric acid Cat. No. C9008, gallic acid Cat. No. G7384, caffeine Cat. No. C0750). Compounds were dissolved in distilled water at the desired concentration (over moderate heat if necessary) and 20% sucrose (*w*/*v*) was then mixed thoroughly into the solution. Each syringe containing one concentration of one phytochemical mixed in 20% aqueous sugar solution was provided to the bees to feed *ad libitum*. The feed solutions were replaced every 10–12 days to maintain freshness. The cages were monitored daily, date of mortality and number of dead bees recorded, and the dead bees removed.

### 2.2. Pathogen Tolerance Assay

*Nosema ceranae* spores were obtained from Naug lab at Colorado State University where previous studies used multiplex PCR and electrophoresis to confirm the species [44,45]. The spores were in the form of macerated intestinal tract suspension. Initial spore concentration was determined by counting using a hemocytometer [46]. To generate fresh spore inoculum for this assay, the spore suspension mixed with 50% sucrose solution was fed to the worker honey bees that were maintained in cages for a week. This procedure has been shown to be effective in generating a new infection [46]. Guts of infected bees were extracted, and the spores were then re-suspended in 50% sucrose solution to produce a spore inoculum with a concentration of 1 × 10^5^ spores per μL required to feed 8 × 10^4^ spores per bee. The spore inoculum was stored at 4 °C and used for inoculation in the coming weeks [46,47,48]. 

Bees were starved for two hours before individual feeding with experimental and control inoculum [44]. Individual feeding was used to ensure exposure to a known quantity of spores, and to produce lower variation in infection level between bees [49]. Eight-day-old bees were force fed 2 µL of spore inoculum by placing the inoculation droplet against the mouthparts of the bee until the entire droplet was consumed. This process is regularly used for artificial infection of honey bees with *N. ceranae* [46]. Spore inoculum fed bees were transferred to single-use cup cages such that there were ten infected bees per cup cage. The cup cages were then randomly allocated to different phytochemical treatments such that there were three cup cages per treatment totaling 30 bees per treatment (Table 1). Three similar cup cages with ten bees per cage were set up as the controls, totaling 30 infected bees receiving 20% sucrose solution. The cages were monitored daily for dead bees that were removed. Spore load in each dead bee was quantified on a hemocytometer after homogenizing the entire gut [46,49]. 

### 2.3. Statistical Analysis

All statistical analyses were performed using IBM Statistics SPSS 25. Kaplan-Meier survival probability estimates were used to evaluate the differences in honey bee survival between phytochemical treatments and concentrations. Log-rank (Mantel-Cox) tests were used for pairwise comparisons. *Nosema* spore loads were log transformed for normality prior to completing the Univariate Analysis of Variance followed by Tukey’s post-hoc comparisons. Longevity of infected bees in the phytochemical supplemented treatment was compared by the Medians test.

## 3. Results

### 3.1. Survival

**Figure 1:**
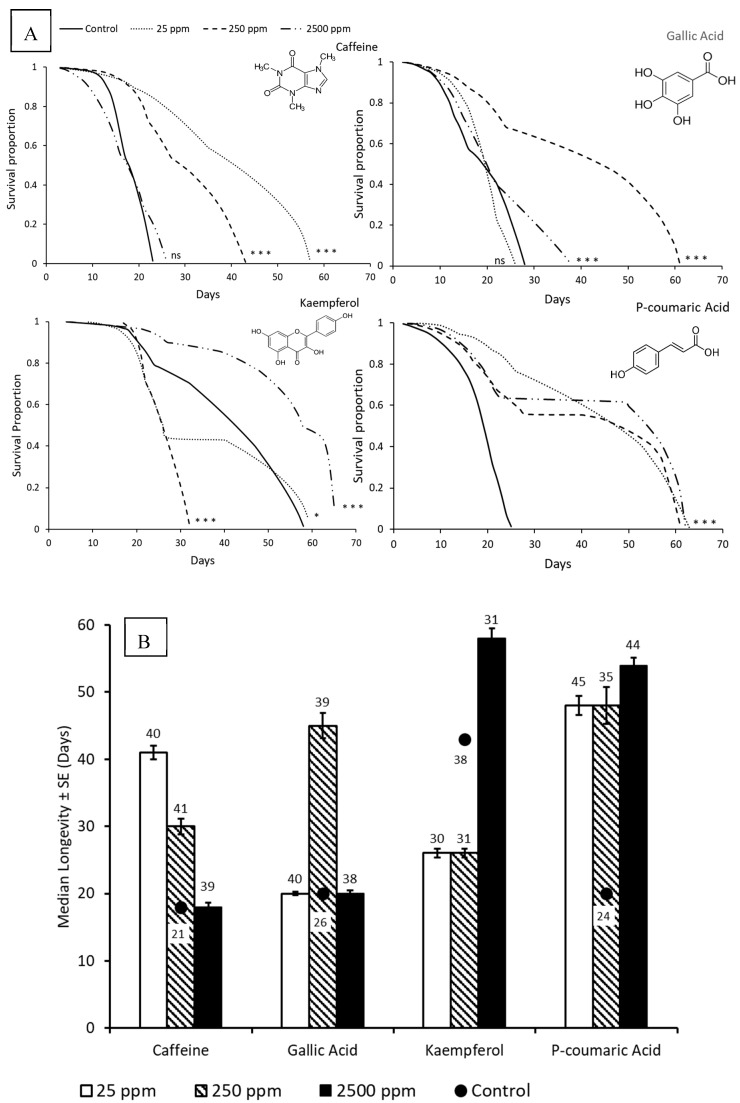
Survival proportions (**A**) and median longevity (**B**) of bees fed *ad libitum* with sucrose solutions supplemented with phytochemicals at different concentrations. Kaplan Maier Survival Analyses and log-rank (Mantel-Cox) pairwise comparisons were used to compare survival rates between Control and different concentrations within each phytochemical compound (*p* < 0.0001 is denoted ***; *p* = 0.01 is denoted by *; *p* > 0.5 is denoted by ns; numbers indicate sample sizes (above treatment bars and below ● respectively). Pairwise comparisons between compounds and doses are provided in Supplement S1.

Worker bees supplemented with dietary phytochemicals survived longer than control bees (Figure 1A) across 2016 and 2017 with no significant effect of years. Lower concentrations of dietary phytochemicals were generally more beneficial (Figure 1B) although effects of phytochemicals on longevity did vary with concentrations. Caffeine at 25 ppm had the highest median longevity, gallic acid had the highest median longevity at 250 ppm and kaempferol supplementation showed highest median longevity at 2500 ppm. In 2016, the effects of different concentrations differed from each other and were all significantly higher than the control, however in 2017, there was no effect of concentrations. Results of pairwise comparison across different concentrations for each of the phytochemicals are presented in Supplement S1 and S2. There was no evidence of the difference in the amount of chemicals and concentrations consumed by the bees as seen by a tolerance assay that measured the amount of the different phytochemicals consumed (Supplement S3 and S4). 

### 3.2. Pathogen Tolerance

**Figure 2:**
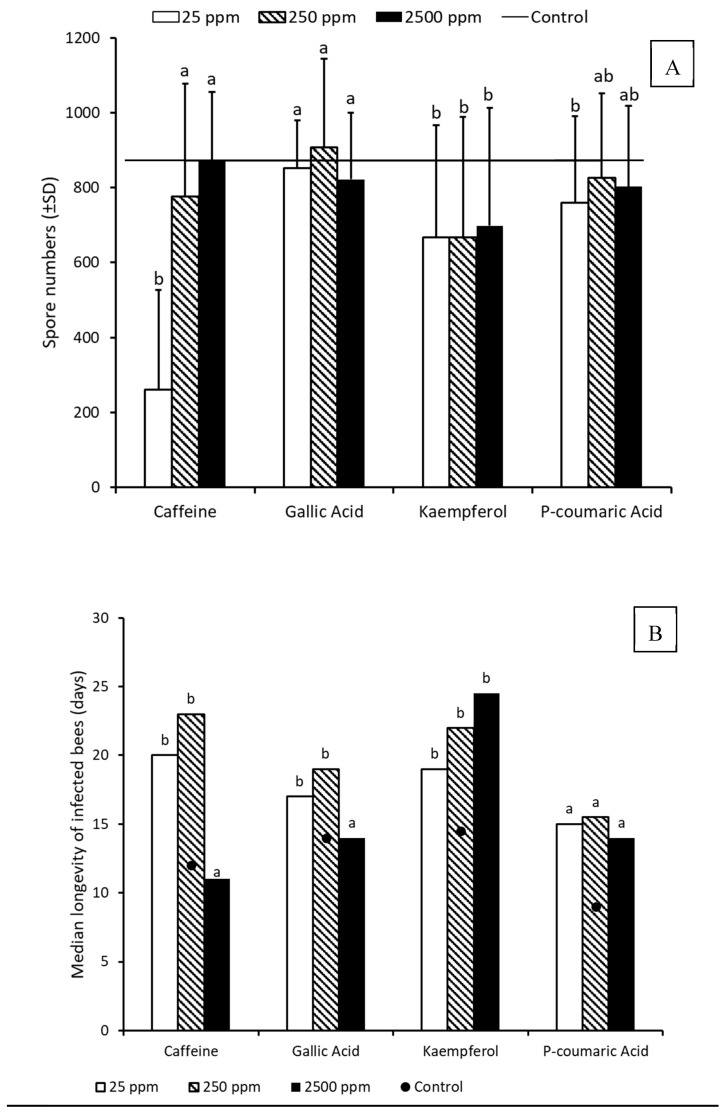
Spore loads (untransformed values) in dead infected bees (**A**) and median longevity of bees (**B**) that received dietary supplementation of phytochemicals at different concentrations (N = 30 bees for each phytochemical treatment; N = 22 for Control). Bars with different letters are significantly different from the control (Spore loads (**A**): GLM Univariate ANOVA followed by Tukey’s post-hoc comparison of log-transformed spore loads; Longevity (**B**): Median test).

Phytochemical supplementation in the diet of bees infected with *N. ceranae* had a significant effect on spore-load at mortality. Results of GLM Univariate ANOVA indicated significant direct effects of phytochemicals and concentrations, no detectable colony effect and a significant interaction effect between the phytochemical and concentrations (Table 2). Post-hoc comparisons indicated that caffeine, kaempferol and *p*-coumaric acid reduced spore-loads significantly as compared with the infected controls (Figure 2A), and low concentration (25 ppm) was most effective in reducing spore-loads (Figure 2A). Comparison of median longevity by the Medians test indicated a significant increase in longevity of infected bees supplemented with caffeine, gallic acid and kaempferol at low (25 ppm) and medium (250 ppm) concentrations (Figure 2B; Supplement S5). While *p*-coumaric acid and kaempferol significantly increased longevity across all concentrations in comparison with the longevity of the infected control bees, the other phytochemicals tested only increased longevity at lower concentrations.

## 4. Discussion

In this study, we showed that phytochemicals promoted worker bee longevity and pathogen tolerance. Previous studies have reported a similar increase in longevity with diets individually supplemented with *p-*coumaric acid, quercetin [8] and caffeine [37], but similar studies exploring the effects of gallic acid and kaempferol are lacking. While concentration-related responses were evident in caffeine, gallic acid and kaempferol, generally lower concentrations were more effective both in promoting pathogen tolerance and increasing longevity. Phytochemicals are plant secondary metabolites that act as toxins and deterrents protecting plants against insect pests and pathogens. Honey bees consume substantial amount of these secondary compounds contained in the pollen, nectar, honey and bee bread stored in colonies [8]. Studies supporting the strength of beneficial co-evolution between floral chemical traits and pollinators are limited [50] but increasing evidence indicates tolerance to naturally occurring compounds in nectar [51]. In other studies, phytochemical concentrations showing benefits were found to be similar to the naturally occurring concentrations in floral nectar [24]. Compounds in honey, such as *p-*coumaric acid and other phenolic acids, similar to the ones reported in our study improve longevity and upregulate detoxifying enzymes [20,52,53], and flavonols, such as quercetin influence longevity and enhance tolerance to certain pesticides [8]. Our results add further evidence to this growing field supporting the idea that plant defense compounds provide benefit to honey bees. 

Insect pollinators specifically those receiving low concentrations, could develop tolerance when toxicity effects are mild. Caffeine, in our studies, increased longevity at 25 and 250 ppm but reduced longevity below that of the control at a high concentration of 2500 ppm. Caffeine at low concentrations has been shown to increase longevity, limit infection from *Nosema* spp., decrease age-related metabolic tendencies and decrease DNA methylation levels in older bees, supporting the recommendation of the use of caffeine for stress-resistance in bee colonies [37]. Benefits of low levels of caffeine in sucrose on learning and retention have been demonstrated [36], where caffeine has a strong effect on long term memory, but a weak effect on the rate of learning. In addition, bees in the study were more likely to reject sucrose with caffeine at higher concentrations. Our tolerance assays, however, did not show any differences in the amount of phytochemicals consumed over a given duration for the concentrations we tested, suggesting that there is no evidence for preference between the different concentrations in our study. Being able to reject sucrose solutions with high concentrations of caffeine [36] has been suggested as a mechanism to drive selection towards floral caffeine concentrations that are not repellent but pharmacologically active. Our results suggest that the detrimental effect of reduced longevity from consumption of 2500 ppm (high concentration) of caffeine could potentially be the selective factor for the learning ability in bees to avoid flowers with high caffeine, while the increased longevity in low and medium concentrations may play an important role in maintaining the pharmacologically active concentrations of caffeine in nectar by promoting learning and memory. Further studies assaying nectar concentrations of phytochemicals are necessary to confirm this hypothesis. Similar studies on the effects of the other phytochemicals tested in our study of bee learning and memory are currently lacking.

Dietary supplementation with caffeine, kaempferol and *p*-coumaric acid at 25 ppm significantly reduced spore-load in infected bees. Kaempferol reduced spore-load at all concentrations while caffeine was not beneficial at higher concentrations. The detrimental effect of high caffeine concentration was more prominent in infected bees as seen by the reduced longevity of infected bees supplemented with 2500 ppm caffeine. Infected bees supplemented with phytochemicals in their diet lived longer than the infected control bees. The control bees in the survival assay that were healthy, showed a median longevity of 20 days as against that of 12 days in the infected control bees. This reduced longevity of the infected control bees is to be expected, but phytochemical supplementation increased the longevity of the infected bees to 23 days (caffeine 250 ppm), slightly higher than even the healthy bees. While it is possible that spores of *N. ceranae* could have moved between bees through trophallaxis, it is unlikely to have solely influenced the final spore count in dead bees as the phytochemicals in the diet would also be shared during the process. Phytochemical supplementation continued to show an increase in longevity but such an increase together with the observation that infected bees receiving phytochemical supplementation had low loads of *N. ceranae* spores, poses an interesting conundrum of whether infection with access to phytochemicals at low concentrations could pose a risk of increased spore transmission. Studies have shown that bees infected with *N. ceranae* that also experience starvation are highly likely to exhibit higher and risky foraging [44]. However, if bees access phytochemicals while consuming floral nectar, the risk of starvation is minimized which could then lead to a reduced likelihood of foraging by the infected bees. This reiterates the importance of floral nectar sources that provide bees with a full complement of dietary compounds and meet the energy demands, promoting the ability of bees to tackle pathogen infection and minimize the spread of pathogens to other hives. 

The consequences of exposure to plant defense chemicals, while costly, may not outweigh the benefits to bees [13,18,54]. Consumption of gelsemine, a nectar alkaloid found in *Gelsemium sempervirens*, lessens the severity of gut pathogen infections in bumble bees [19]. Other studies have demonstrated the functional role of alkaloids, glycosides, phenolics, and terpenoids in reducing gut pathogen load in artificially infected bumble bees [18]. A number of poisonous plant genera require their associated pollinators to adopt biochemical, physiological, and behavioral mechanisms to cope with toxic co-occurring phytochemicals [55,56]. Dietary diversity as in a diverse array of plant species, as well as the diversity of phytochemicals within floral nectar, are primary factors in honey bee immuno-competence. Polyfloral pollen promotes individual and social immunity [57] and the resulting phytochemical diversity from polyfloral pollen can also play an important role in honey bee immune responses [52]. While there are not many studies that describe the phytochemical composition of nectar and pollen of common flowering plants [23], a few studies indicate the presence of caffeine in the nectar of *Coffea* spp. and *Citrus* spp., coumaric acid, coumarin and other derivatives in *Melilotus* spp., gallic acid in flowers of some plants from Malvaceae and Ericaceae families, and kaempferol in plants of Ericaceae family [12,22,32,36,58]. High levels of floral fidelity demonstrated by honey bees, may pose challenges to acquiring an adequate mixture of phytochemicals. However, the colony allocates its large foraging force such that only a subset of workers may be recruited to a single reward patch even as other groups of foragers explore other patches allowing the colony to gather an array of different phytochemicals. Availability of these floral chemicals is dependent upon the bees being able to access polyfloral nectar and pollen. Our results reiterate the importance of diverse flowering habitats because increasing monocultures and loss of wildflowers negatively affect natural mutualistic relationships [1,59]. 

## 5. Conclusions

The complex mutualistic interactions are highly suggestive of the functional significance of phytochemicals leading to profound implications for honeybee health and colony management practices. Extending the worker bee lifespan increases the overall foraging time allowing the colony to rapidly collect and store greater amounts of nectar and pollen. The increase in longevity and pathogen tolerance also have positive implications for colony productivity. Supplemental feeding of honey bee colonies is common practice among beekeepers to promote colony growth and overall health. While experimental evidence exists for the benefits of protein and carbohydrate supplementation in increasing the probability of colony survival, it is clear that supplementation with sugar is not equivalent of bees consuming honey [52] which is a mixture of polyfloral nectar and phytochemicals therein. As supplemental feeding becomes a necessity with dwindling natural habitats, adding phytochemicals to the supplements is likely to become a crucial necessity. For the future, colony level studies on the benefits of phytochemicals would provide valuable data on the significance of these phytochemicals.

## Figures and Tables

**Table 1:** Structures and classification of phytochemicals used for dietary supplementation.

Phytochemicals	Classification	Chemical Structure
Caffeine	Alkaloid	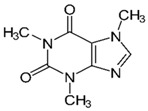
Gallic acid	Flavonol	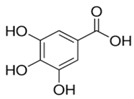
Kaempferol	Phenolic acid	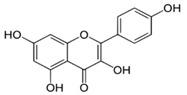
*p*-coumaric acid	Phenolic acid	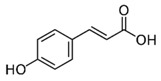

**Table 2:** GLM Univariate analysis of *N. ceranae* spore load in worker bees with dietary supplementation of phytochemicals at different concentrations. Bold cells indicate significance.

Source of Variation	df	MSS	F	*p*
*Direct effects*				
**Model**	**1**	**3586.66**	**166,225.64**	**<0.0001**
**Phytochemical**	**3**	**1.53**	**24.61**	**<0.0001**
**Concentrations**	**2**	**1.32**	**14.01**	**0.002**
Colony	4	0.02	0.23	0.9
*Interaction effects*	6			
**Phytochemical × Concentration**	**6**	**1.15**	**16.92**	**<0.0001**
Phytochemical × Colony	12	0.06	0.91	0.5
Concentration × Colony	8	0.09	1.39	0.3
Phytochemical × Concentration × Colony	24	0.07	1.35	0.13

## Supplementary Materials

The following are available online at http://www.mdpi.com/2075-4450/10/1/14/s1, Figure S1: Average diet consumed for each of the phytochemical treatment and the control solutions. Figure S2: Survival proportions of bees infected with Nosema ceranae spores and then fed ad libitum with sucrose solutions supplemented with phytochemicals at different concentrations. Kaplan Maier Survival Analyses were used to compare survival rates. Table S1: The effect on longevity elicited by different concentrations of the same phytochemical. Kaplan-Maier survival analyses followed by Log rank (Mantel Cox) comparisons, Table S2: The effect on longevity elicited by different phytochemicals at similar concentrations (Conc.) Kaplan-Maier survival analyses followed by Log rank (Mantel Cox) comparisons. Methods S1: Tolerance assay to measure the amount of phytochemicals consumed by bees.
