# Supplementary material for: Dietary Phytochemicals, Honey Bee Longevity and Pathogen Tolerance
Source: Insects. 2019 Jan 8;10(1):14. doi: 10.3390/insects10010014 (PMC6359238; doi:10.3390/insects10010014)

Supplements

Table S1: The effect on longevity elicited by different concentrations of the same phytochemical. Kaplan-Maier survival analyses followed by Log rank (Mantel Cox) comparisons.

Phytochemical	Concentrations	0 ppm		25 ppm		250 ppm		2500 ppm	
		χ^2	p	χ^2	p	χ^2	p	χ^2	P
Caffeine	0 ppm								
	25 ppm	**379.2**	**<0.0001**						
	250 ppm	**250.8**	**<0.0001**	**126.8**	**<0.0001**				
	2500 ppm	1.6	0.21	**712.8**	**<0.0001**	**384.5**	**<0.0001**		
Gallic Acid	0 ppm								
	25 ppm	0.5	0.5						
	250 ppm	**270.1**	**<0.0001**	**268.9**	**<0.0001**				
	2500 ppm	**19.5**	**<0.0001**	**11.1**	**.001**	**351.7**	**<0.0001**		
Kaempferol	0 ppm								
	25 ppm	**7.7**	**0.01**						
	250 ppm	**117.7**	**<0.0001**	**8.9**	**.003**				
	2500 ppm	**219.2**	**<0.0001**	**193.9**	**<0.0001**	**398.8**	**<0.0001**		
p-coumaric acid	0 ppm								
	25 ppm	**603.6**	**<0.0001**						
	250 ppm	**215.5**	**<0.0001**	**15.5**	**.0001**				
	2500 ppm	**248.6**	**<0.0001**	0.4	0.5	**8.9**	**.003**		

Table S2: The effect on longevity elicited by different phytochemicals at similar concentrations (Conc.). Kaplan-Maier survival analyses followed by Log rank (Mantel Cox) comparisons

Conc.	Phytochemicals	Caffeine		Gallic Acid		Kaempferol		*p*-coumaric Acid	
		χ^2	P	χ^2	P	χ^2	p	χ^2	p
25 ppm	Caffeine								
	Gallic Acid	**606.5**	**<0.0001**						
	Kaempferol	**5.9**	**0.02**	**196.01**	**<0.0001**				
	p-coumaric Acid	**35.7**	**<0.0001**	**523.9**	**<0.0001**	**25.4**	**<0.0001**		
250 ppm	Caffeine								
	Gallic Acid	**55.1**	**<0.0001**						
	Kaempferol	**12.9**	**0.0003**	**20.99**	**<0.0001**				
	p-coumaric Acid	**26.3**	**<0.0001**	0.01	0.9	**3.9**	**0.05**		
2500 ppm	Caffeine								
	Gallic Acid	**41.4**	**<0.0001**						
	Kaempferol	**987.6**	**<0.0001**	**1127.8**	**<0.0001**				
	p-coumaric Acid	**295.2**	**<0.0001**	**333.8**	**<0.0001**	**128.4**	**<0.0001**		

Methods S3: Tolerance assay to measure the amount of phytochemicals consumed by bees

The four phytochemicals used for supplementation at different concentrations were set up in individual cup cages (Fig. S3). Each cup cage has three holes of 1 cm diameter each. Two of the holes held 1.5 mL Eppendorf tubes (a feeder tube and a water tube). The third hole was for ventilation and was covered by a black mesh netting that was glued over the hole. The cage bottom was a clear plastic petri dish with a square 10 x 10 cm absorbent liner.

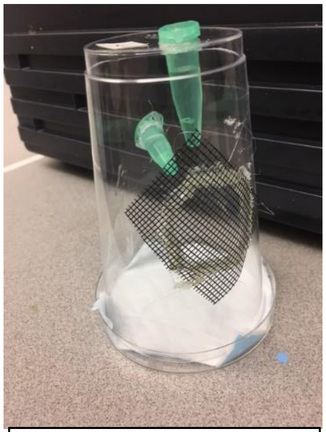

Figure S3: Cup cage setup for the tolerance assay

Five random worker bees were placed in each cage. Each cage had one Eppendorf tube filled with 2.3 grams of the feeding solution (phytochemicals at the 3 doses (Table 1) or 20% sucrose control solution) and the other Eppendorf tube was filled with distilled water. The feeder tubes were weighed before being placed in the cage to obtain the start weight value. Bees in their cages were kept in an incubator at 34°C and 40% humidity. Each day the end weight value of each feeder tube was recorded, number of bees still alive for each treatment noted and any dead bees removed. After recording the end weight value, the feeder tubes were refilled, weighed and reinserted into the cage. On day 4, the end weight value for each feeder and number of bees still alive were noted for each cage and the cages were disassembled. A total of fourteen cup cages were used for the trials – three for each of the four concentrations and two for the control. Two cages were set up with Eppendorf tubes filled with the same concentrations to record evaporation.

Amount consumed = ((Start weight – end weight) – evaporation value)) averaged over the number of bees alive each day.

The average amount consumed over the 3 day trial period (Fig. S4) was used for one-way ANOVA for each of the phytochemical treatment.

Figure S4: Average diet consumed for each of the phytochemical treatment and the control solutions

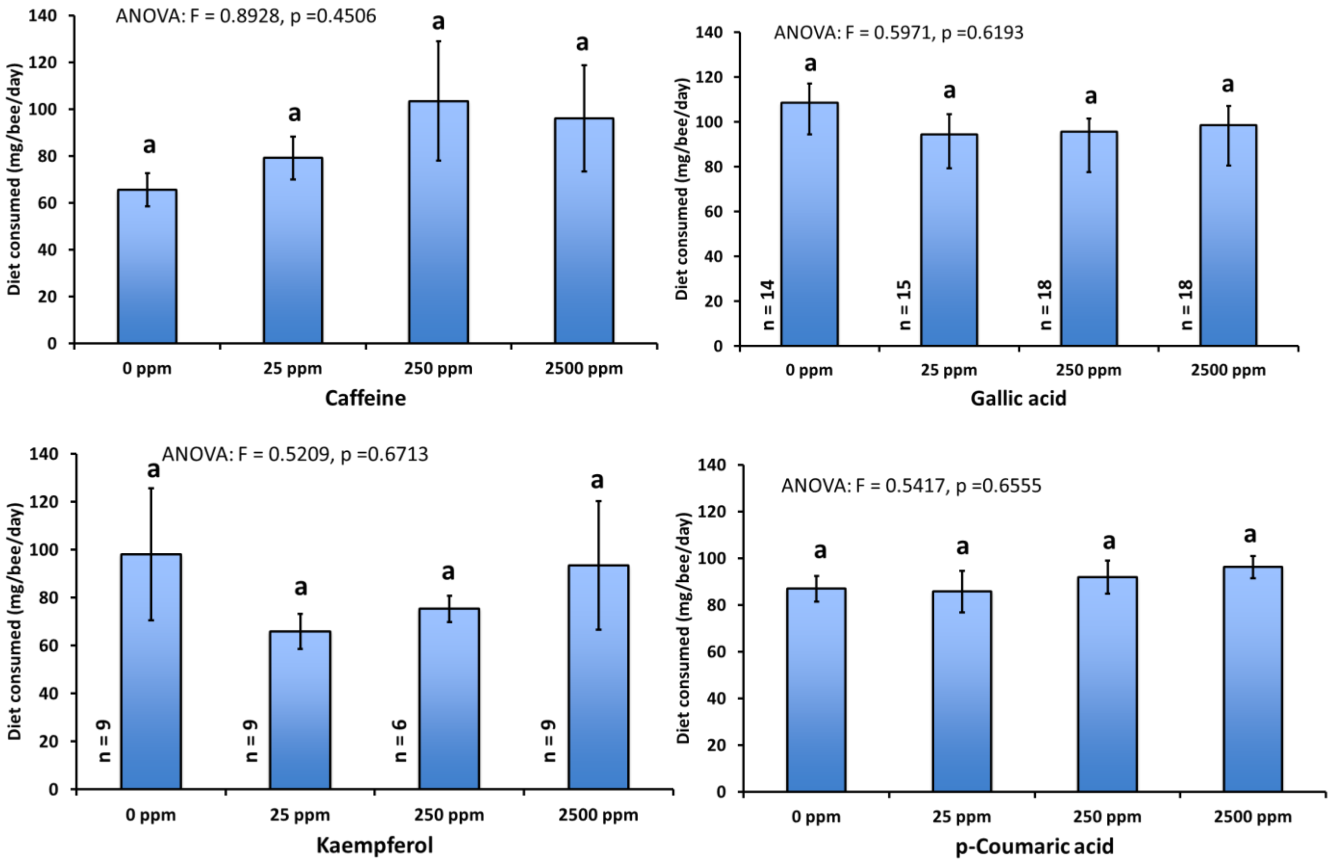

Figure S5: Survival proportions of bees infected with *Nosema ceranae* spores and then fed *ad libitum* with sucrose solutions supplemented with phytochemicals at different concentrations. Kaplan Maier Survival Analyses were used to compare survival rates.

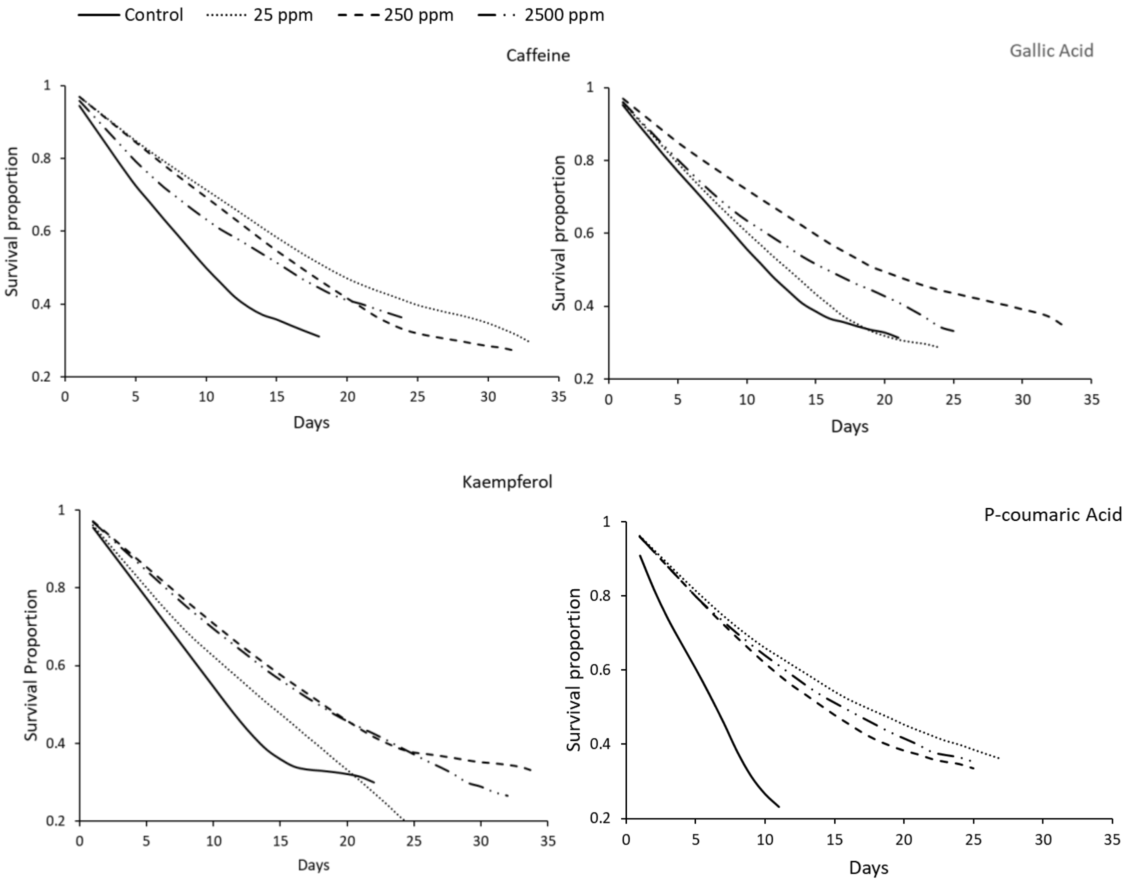

Supplement: Supplementary file 1 [file insects-10-00014-s001.pdf]
